# Retrospective observational analysis of esophageal foreign bodies: a novel characterization based on shape

**DOI:** 10.1038/s41598-020-61207-8

**Published:** 2020-03-06

**Authors:** Wei-Shuyi Ruan, Yu-Ning Li, Meng-Xiao Feng, Yuan-Qiang Lu

**Affiliations:** 1 Department of Emergency Medicine, The First Affiliated Hospital, School of Medicine, Zhejiang University, Hangzhou, 310003 Zhejiang People’s Republic of China; 2 Department of Geriatric Medicine, The First Affiliated Hospital, School of Medicine, Zhejiang University, Hangzhou, 310003 Zhejiang People’s Republic of China; 3 Zhejiang Provincial Key Laboratory for Diagnosis and Treatment of Aging and Physic-chemical Injury Diseases, The First Affiliated Hospital, School of Medicine, Zhejiang University, Hangzhou, 310003 Zhejiang People’s Republic of China; 4 School of Mathematical Sciences, Zhejiang University, Hangzhou, 310058 Zhejiang People’s Republic of China

**Keywords:** Oesophagogastroscopy, Risk factors

## Abstract

This single-center retrospective study aims to investigate the clinical features of esophageal foreign bodies (EFBs) and determine the influence of EFB shapes on management and prognosis. A total of 427 patients aged 13 to 95 years with suspected EFB ingestion were enrolled between January 2013 and June 2018, 183 of whom were male. EFBs were divided into six shapes: pin (n = 161), sheet (n = 97), trident (n = 51), spindle (n = 66), irregular (n = 46), and sphere (n = 6). Spindle-shaped EFBs correlated with a significantly higher rate of perforation and severe complications (*P* < 0.001 and *P* = 0.021, respectively) than any other EFB shape, while sheet-shaped EFBs were linked to less severe complications (*P* = 0.006). The number of pressure points was provided to stratify the risk of poor prognosis for each shape. EFBs with only two pressure points (pin and spindle EFBs) required more advanced management strategies and were correlated with a higher number of patients suffering esophageal perforation (27.11%) and severe complications (12.44%) when compared with other shapes (χ^2^ = 11.149 and *P* = 0.001; *χ*^2^ = 5.901 and *P* = 0.015, respectively). Spindle shape was an independent risk factor for poor prognosis, and contributed a more clinical risk than the pin shape. In conclusion, clinical features, management, perforation rate, and severe complications differed based on EFB shape. The EFBs with two pressure points, especially the spindle-shaped EFBs, were more dangerous compared with those with more pressure points.

## Introduction

An esophageal foreign body (EFB) is a relatively common complaint in the emergency room. Patients often complain about dysphagia, retrosternal pain, and occasionally abdominal pain prior to being diagnosed with an EFB. An EFB is often accompanied by serious medical conditions, such as cervical abscess, mediastinitis, aortoesophageal abscess, tracheoesophageal fistula, pneumonia, and pneumothorax, most of which are caused by esophageal perforation^1–6^. Thus, an EFB can lead to death if the diagnosis is significantly delayed. However, some patients who have ingested foreign bodies do not immediately go to the hospital; instead, they attempt to dislodge EFBs, especially fish bones, by swallowing rice or vinegar^7^. This prolongs the time between ingestion and effective treatment and leads the injury to become worse. Thus, it is important to appropriately evaluate the location, size, and shape of the foreign body and provide treatment in an emergency medical situation^8^. Types of EFBs may differ among countries and regions according to eating habits, food culture, and sociocultural characteristics^9,10^. In Asian countries, fish bones are the most frequent cause of EFBs; whereas, in Western countries, impacted meat is prevalent^11^. In addition, many studies report interesting cases of patients ingesting unusual EFBs. For instance, Walton encountered a case that presented with torticollis after ingesting a button battery^12^, and Agrawal reported a case that ingested a metallic magnet with sharp metallic hooks on its surface^13^.

Generally, an EFB diagnosis is based on patient history and an examination; doctors examine the oral cavity and laryngopharynx through a laryngoscope when they suspect EFB impaction. According to the European Society of Gastrointestinal Endoscopy (ESGE)^8^, plain radiography is recommended to assess the presence, location, size, configuration, and number of EFBs if the ingestion of radiopaque objects is suspected or the type of object is unknown, and a computed tomography (CT) scan is recommended in all patients with suspected perforation or other complications. Marco performed a prospective study to compare the utility of CT with that of barium swallow and concluded that CT is easier, faster, and more sensitive than barium swallow. Though barium swallow is the primary radiologic method, it may involve a risk of aspiration and can impede a subsequent esophagoscopy^14^, which is consistent with the recommendations of the American Society for Gastrointestinal Endoscopy and ESGE^8,15^. Endoscopy is a major treatment for removing EFBs, of which there are two popular types: flexible endoscopy (FE) and rigid endoscopy (RE). Most experts consider FE the first-line therapy due to its high success rate, but some cases still fail or are not suitable for removing EFBs; in such cases, RE is used as second-line therapy^16^. Surgery is considered a last resort and is usually reserved for high risk cases in which severe complications are suspected.

Furthermore, the ESGE guidelines recommend emergent therapeutic esophagogastroduodenoscopy, preferably within two hours of ingestion, for foreign bodies inducing complete esophageal obstruction and for sharp-pointed objects in the esophagus. Moreover, the guidelines suggest using suitable extraction devices according to the type and location of the EFB. The ESGE guidelines classify five types of EFBs: blunt objects, sharp-pointed objects, long objects, food bolus, and others. Sharp-pointed objects comprise fine objects (e.g., needles, toothpicks, bones, safety pins, and glass pieces) and sharp irregular objects (e.g., partial dentures and razor blades). In China, sharp-pointed EFBs are the most common. For example, jujube pits as jujubes are widely consumed in China. Although bones, jujube pits, and glass pieces are all sharp-pointed, they are shaped differently. Fish bones can be pin-like and trident (Y-shaped), with two to three sharp-pointed ends; jujube pits are spindle-shaped with two sharp-pointed ends and an oval body. Sharp irregular objects and glass pieces can be diverse in shape with three or more sharp-pointed ends. Sharp ends, which also comprise the pressure points in most circumstances, will share the force exerted by the esophageal wall; thus, the pressure on each sharp end will be negatively correlated with the number of ends, which may be related to the risk of EFB. Therefore, the clinical features, management, and prognoses for these shapes of EFBs vary, depending on the number of pressure points. No guidelines and few statistical studies distinguish the clinical risk of differently shaped EFBs. What shape incurs the most risk? Do all sharp-pointed EFBs require the same medical attention regardless of the number of sharp-pointed ends? In this study, we divide EFBs into different groups based on their shapes, investigate the clinical characteristics of each shape and their impact on patients’ management and prognoses, and stratify the risk of each shape according to the number of pressure points.

## Data and Methods

### Inclusion and exclusion criteria

**Figure 1:**
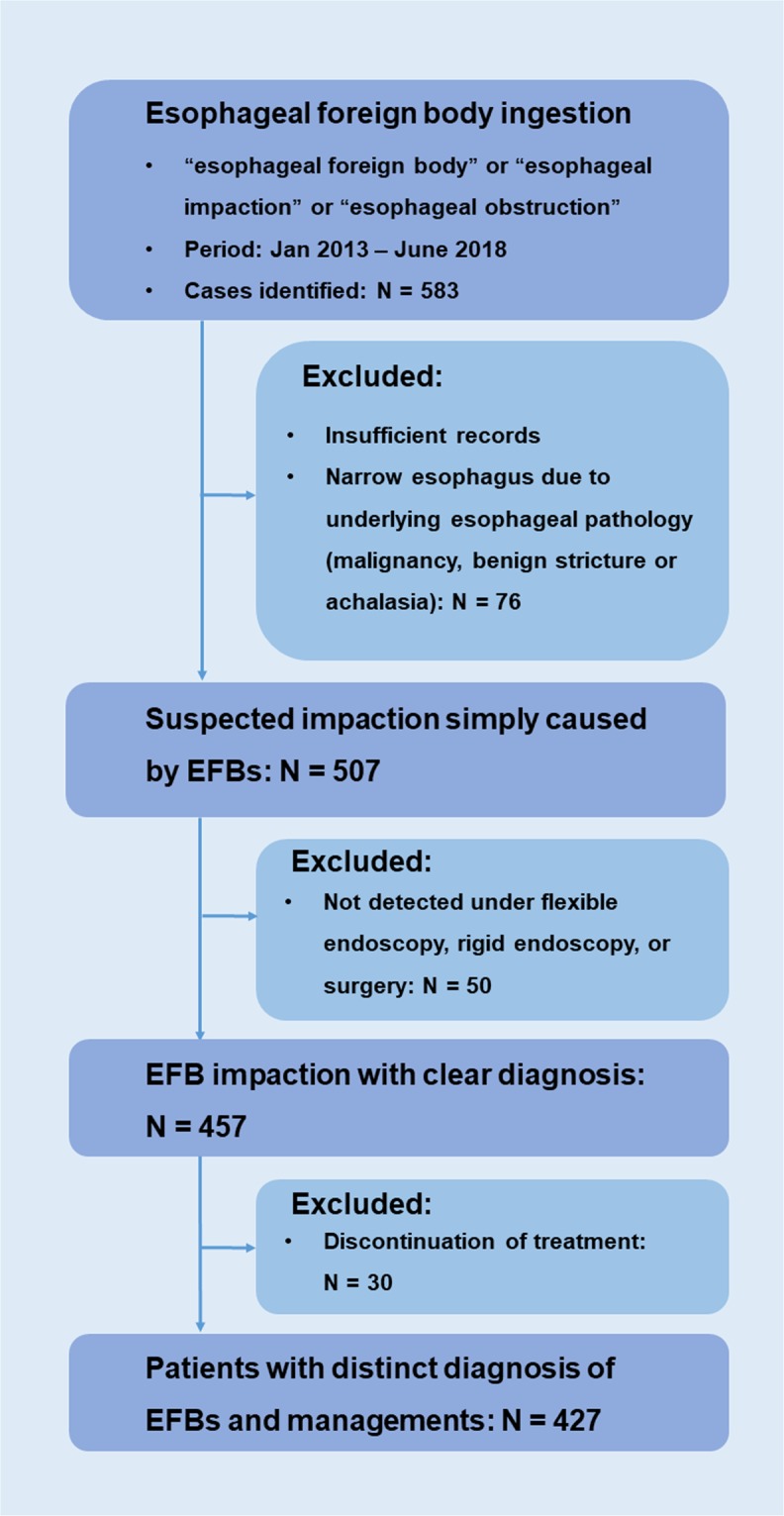
Enrollment of patients.

Data were derived from the electronic medical record system of the First Affiliated Hospital, School of Medicine, Zhejiang University. All 583 cases with suspected EFB ingestion between January 2013 and June 2018 were enrolled. Cases were identified by reviewing the consultation and hospitalization records that contained a diagnosis of EFB, esophageal impaction, or esophageal obstruction and in which the foreign bodies were confirmed by FE, RE, or surgery. After excluding 76 cases with insufficient records or a narrow esophagus due to underlying esophageal pathology (malignancy, benign stricture, or achalasia), 50 cases without detected EFBs under FE, RE, or surgery, and 30 cases in which treatment in this hospital was discontinued (patients who denied subsequent management suggested by physicians and were discharged or admitted to other hospitals), 427 patients were included for analysis (Fig. 1).

### Data extraction

The following records were individually reviewed by two experts to identify types of foreign bodies, demographics, and clinical data, including the type, shape, size, and location of the EFB; the sharpness of the edges; age; gender; chief complaints; duration of impaction (DOI); management; length of stay (LOS); perforation; other severe complications; death. For radiopaque objects, we estimated the shape based on the photos taken under endoscopy or surgery combined with the CT scan and three-dimensional reconstruction. For radiolucent objects, the photos were the only evidence of an EFB, but only one case that ingested a part of a leaf had a negative CT scan in this study. Although it was difficult to consistently categorize all EFB shapes, we nevertheless determined five common shapes that described most of the cases in this study: pin, sheet, trident, spindle, and sphere. The EFBs that were difficult to define were classified as irregularly shaped. The sharp ends of EFBs were recognized as the pressure points. EFB types were defined and divided into the following nine groups: fish bones, poultry bones, pork bones, bones of other animals (frogs, turtles, etc.), jujube pits, dentures, metals, plastics, and other types (food boluses, toothbrushes, herbs, etc.). The sizes of the EFBs were measured as the length of the major axis (Lmax) and the length of the minor axis (Lmin). EFBs with sharp edges, pointed ends, or sharp hooks were classified as sharp EFBs. The location of each EFB was measured as the distance of the EFB from the incisor (DFI). The distance between each EFB and the aortic wall was measured for cases with a high risk of aortoesophageal injury. DOI was defined as the time between ingestion and effective removal. Perforation was suggested by clinical symptoms and typical CT presentations (EFBs penetrating outside the esophagus, periesophageal air, or inflammation around the EFBs) and confirmed under endoscopy or during surgery.

### Statistical analysis

Data were analyzed with SPSS software (version 21.0; IBM Corp., Armonk, NY, USA) and R-project (version 3.3.0 for Windows 32 bit). A *t*-test was used to analyze normally distributed variables, and a Mann–Whitney U-test was used to analyze non-normally distributed variables. A chi-squared test and Fisher’s exact test were used to assess the differences for categorical variables. The Spearman rank correlation was used to investigate relationships among variables through the PerformanceAnalytics package. The Ggplot2 package was used to plot the estimated probability distribution of the impaction location via kernel density estimation (the Y-axis represented probability density, and the X-axis represented DFI). Statistical significance was designated by a *P* < 0.05.

### Ethics approval and Informed consent

The study protocol was reviewed and approved by the Ethical Committee of the First Affiliated Hospital, School of Medicine, Zhejiang University (Reference Number: 2018386). We obtained written informed content for all enrolled patients. The treatment protocols were carried out in accordance with the principles of the Helsinki Declaration.

## Results

### Baseline characteristics

#### Characteristics of EFBs

Table 1 shows that animal bones were the most common EFB, including fish, poultry, pork and other animal bones. Other types of EFBs were jujube pits, dentures, metals, plastics, etc. According to the table, fish bones were primarily pin-shaped; poultry and pork bones were often sheet-shaped; jujube pits were all spindle-shaped; dentures were always irregular-shaped; and others, like food bolus, were mostly sphere-shaped. Four-hundred nine cases ingested sharp EFBs. Only 18 cases ingested blunt EFBs, including six poultry bones (one pin-shaped, two sheet-shaped, two trident-shaped, and one irregular-shaped), four pork bones (one pin-shaped and three sheet-shaped), one metal (sphere-shaped), one plastic (sheet-shaped), and six other types (one sheet-shaped and five sphere-shaped). All sphere-shaped EFBs were blunt (*P* < 0.001).

**Table 1:** Baseline characteristics of esophageal foreign bodies.

Parameter	Total cases	Shape						**P**
		Pin (n = 161)	Sheet (n = 97)	Trident (n = 51)	Spindle (n = 66)	Irregular (n = 46)	Sphere (n = 6)	
Gender (male/female)	183/244	63/98	52/45	25/26	16/50	25/21	2/4	0.003**
Age (y)	52.32 ± 17.72	52.72 ± 14.63	47.43 ± 20.39	46.31 ± 16.91	63.39 ± 15.44	51.83 ± 18.02	53.50 ± 24.04	0.001**
DOI (h)	27.04 ± 44.12	28.09 ± 49.21	20.13 ± 37.18	24.37 ± 34.57	39.89 ± 50.43	21.98 ± 36.90	30.17 ± 33.36	0.309
DFI (cm)	20.34 ± 4.81	20.52 ± 5.38	21.66 ± 4.10	19.49 ± 3.69	18.02 ± 2.76	20.74 ± 5.90	23.50 ± 6.92	<0.001***
LOS (day)	2.55 ± 3.34	2.51 ± 2.99	2.29 ± 2.34	1.90 ± 1.81	3.12 ± 2.89	3.09 ± 6.67	2.67 ± 2.42	0.816
Lmax (cm)	22.15 ± 9.01	21.10 ± 8.20	22.46 ± 6.65	21.21 ± 9.87	20.85 ± 8.11	26.83 ± 13.20	31.83 ± 11.99	0.010*
Lmin (cm)	9.17 ± 6.87	3.40 ± 1.28	15.91 ± 5.66	11.60 ± 6.77	7.08 ± 2.42	14.01 ± 6.45	20.00 ± 4.86	<0.001***
Lmax/Lmin	3.95 ± 3.14	6.81 ± 3.12	1.53 ± 0.57	2.53 ± 2.28	3.05 ± 0.84	2.22 ± 1.36	1.58 ± 0.48	<0.001***
Sharpness (sharp/blunt)	409/18	159/2	90/7	49/2	66/0	45/1	0/6	<0.001***
Types								
Fish bone	198(46.37%)	121	37	23	0	17	0	/
Poultry bone	113(26.46%)	33	41	23	0	16	0	/
Pork bone	21(4.92%)	4	15	2	0	0	0	/
Other animal bone	7(1.64%)	3	2	2	0	0	0	/
Jujube pit	66(15.46%)	0	0	0	66	0	0	/
Denture	11(2.58%)	0	0	0	0	11	0	/
Metal	3(0.70%)	0	0	1	0	1	1	/
Plastic	2(0.46%)	0	1	0	0	1	0	/
Others	6(1.41%)	0	1	0	0	0	5	/

DOI: duration of impaction; DFI: distance from incisor; LOS: length of stay; Lmax: length of major axis; Lmin: length of minor axis. *P < 0.05; **P < 0.01; ***P < 0.001.

#### Clinical features

One-hundred eighty-three men (42.86%) and 244 women (57.14%) were included in this study. The gender distribution was significantly different among different shapes of EFBs (*P* = 0.003); most female patients ingested pin-shaped (n = 98) and spindle-shaped EFBs (n = 50). The mean age was 52.32 ± 17.72 years (range 13–95 years), and was the oldest in the spindle group, and youngest in the trident group (*P* = 0.001). The mean DFI was 20.34 ± 4.81 cm and was the shortest in the spindle group and largest in the sphere group (*P* < 0.001), which means spindle-shaped EFBs impacted the upper esophagus more frequently than any other shape, and sphere-shaped EFBs impacted the lower esophagus. The mean DOI was 27.04 ± 44.12 hours, and the mean LOS was 2.55 ± 3.34 days. No significant differences in DOI or LOS were found among the groups. The mean Lmax was 22.15 ± 9.02 mm (range 4.5–70 mm), and it was the shortest in the spindle group and longest in the sphere group (*P* = 0.010). The mean Lmin was 9.17 ± 6.87 mm (range 2–31 mm); it was shorter in the pin and spindle groups than that of the other groups (*P* < 0.001). The Lmax/Lmin ratio of the pin group was the largest of all the groups, followed by that of the spindle group (*P* < 0.001). The mean Lmax and Lmin of sharp EFBs were 27.22 ± 7.92 mm and 15.42 ± 2.02 mm, respectively, and longer than those of blunt EFBs (21.93 ± 9.00 mm and *P* = 0.015; 8.89 ± 4.04 mm and *P* < 0.001, respectively). The Lmax/Lmin ratio of sharp EFBs (4.04 ± 3.17 mm) was significantly larger than that of blunt EFBs (2.01 ± 0.88 mm, *P* = 0.007).

### The probability density of EFB impaction for each shape based on size

**Figure 2:**
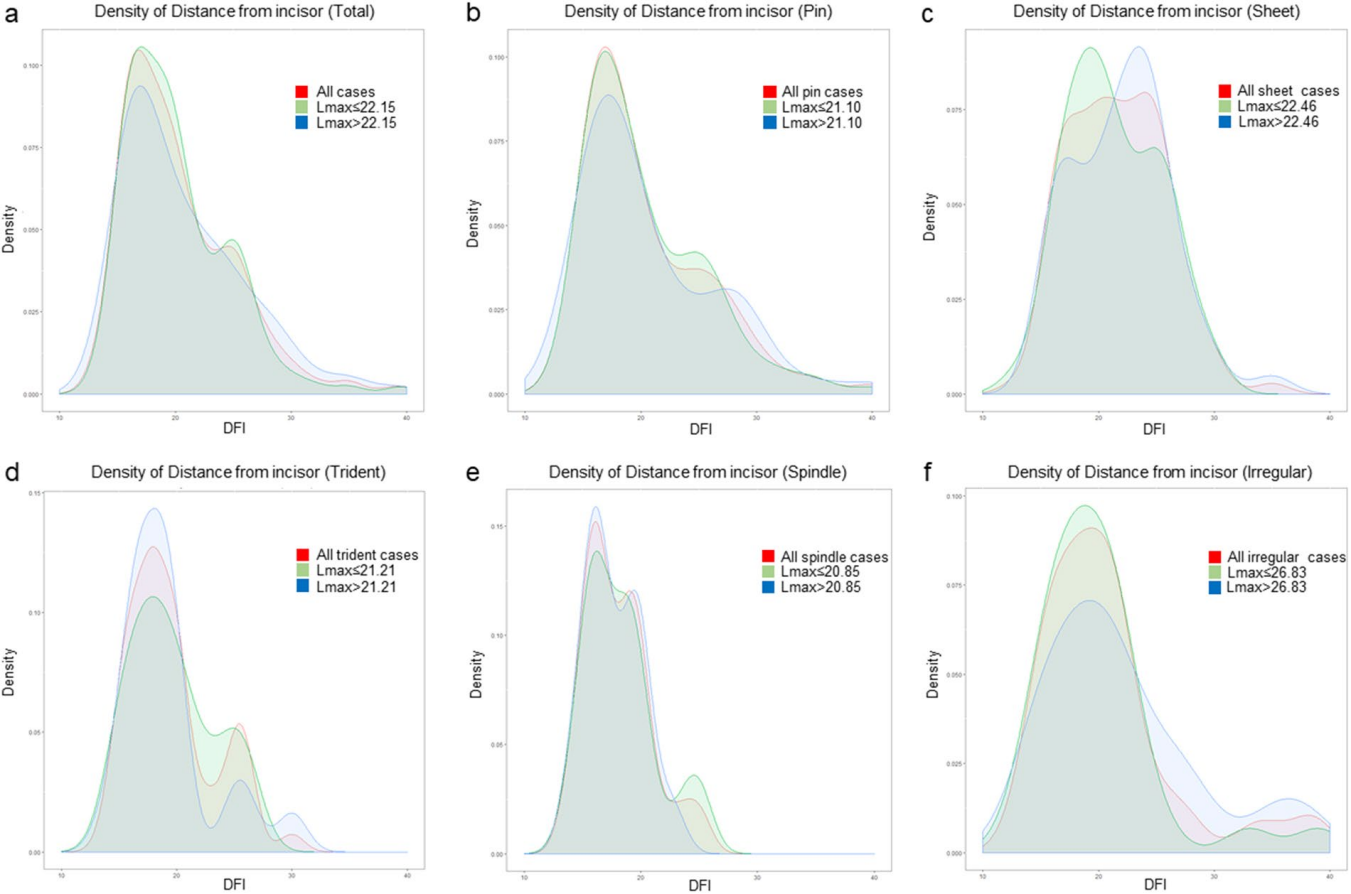
Impacted probability along with DFI. (**a**) For all cases; (**b**) For pin cases; (**c**) For sheet cases; (**d**) For trident cases; (**e**) For spindle cases; (**f**) For irregular cases. DFI: distance of the EFB from the incisor. Lmax: the length of major axis of the EFBs. Red indicates all cases of a specific shape; green indicates the large half of the cases of a specific shape (Lmax > the mean Lmax of the shape); blue indicates the small half of the cases of a specific shape (Lmax ≤ the mean Lmax of the shape).

**Figure 3:**
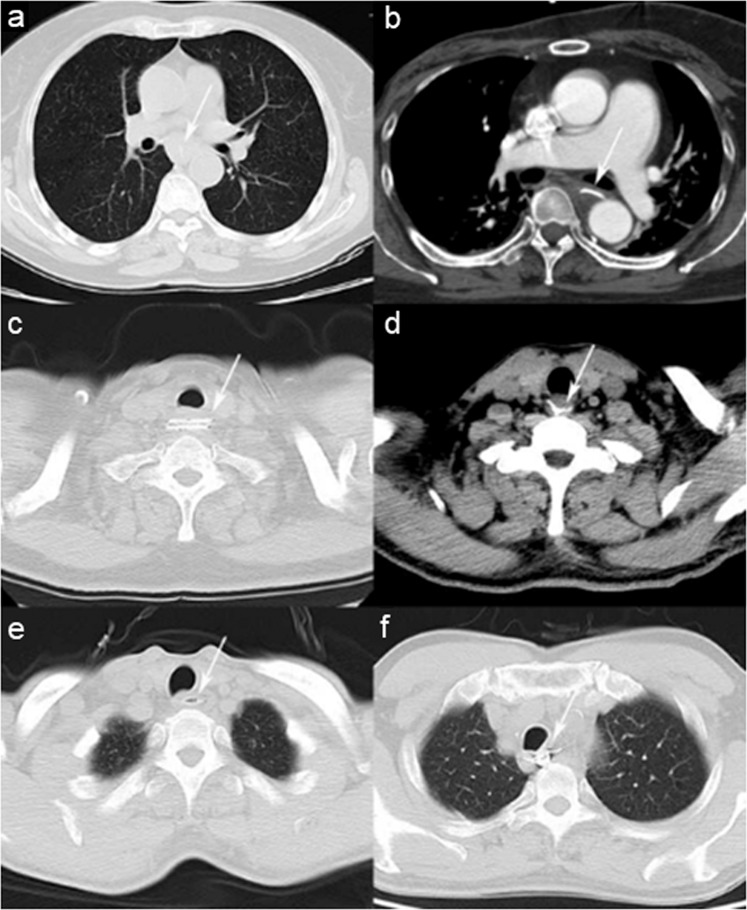
Computed tomographic findings of different types and shapes of esophageal foreign bodies. (**a**) Pin-shaped fish bone lodged in the mid-esophagus in an anterior–posterior orientation. (**b**) Pin-shaped fish bone lodged near the aorta in a left–posterior orientation. (**c**) Sheet-shaped pork bone lodged in the upper esophagus in a left–right orientation. (**d**) Trident-shaped duck bone lodged near the entrance of the esophagus. (**e**) Spindle-shaped jujube pit lodged in the upper esophagus in a left–right orientation. (**f**) Irregular-shaped denture lodged in the upper esophagus.

Figure 2 shows the probability density of EFBs in the upper esophagus compared with that of EFBs in the middle and lower esophagus. The EFBs were divided into the small half and the large half according to the Lmax of the EFBs. The Lmax of the small half of the cases was shorter than the mean Lmax of a specific shape, while the Lmax of the large half of the cases was larger than the mean Lmax of a specific shape. For all cases, the two peak densities were 16.81 cm (10.46%) and 24.56 cm (4.5%), respectively (Fig. 2a); the small half of the cases had similar impaction peak densities (17.10 and 24.89 cm, 10.56% and 4.69%, respectively). These two locations were near the first and second esophageal physiological stenoses. In the large half of all cases, impaction occurred most frequently at 17.00 cm (9.37%) but with less probability than the small half in the upper esophagus. However, the impaction probability of the large half was higher in the middle and lower parts of the esophagus than the small half. The peaks of the impaction location were 16.92 cm (10.30%) and 24.63 cm (3.72%) in the pin group (Fig. 2b); the large half of the pin cases impacted lower than those of the small half (first and second DFI peak: 17.22 cm [8.87%] and 27.34 cm [3.12%], 16.91 cm [10.16%] and 24.65 cm [4.21%], respectively). In the sheet group (Fig. 2c), the impaction location of the majority of cases was between 17.37 and 24.03 cm. The large half of the sheet-shaped cases impacted primarily at 23.50 cm (9.15%), which was substantially lower than that of the small half (19.32 cm, 9.13%). In the trident group (Fig. 2d), the peaks of the impaction location were 17.94 cm (12.73%) and 25.44 cm (5.34%). The large half of the trident cases impacted in the upper esophagus more frequently compared with those of the smaller half (18.06 cm and 14.33%; 17.93 cm and 10.66%, respectively), while in the middle of the esophagus, the impaction probability was reversed (25.52 cm and 3.00%; 24.89 cm and 5.17%, respectively). However, in the lower esophagus (lower than 30 cm), there were almost exclusively large EFBs. In the spindle group (Fig. 2e), the peaks of the impaction locations were 16.14 cm (15.21%) and 19.07 cm (12.03%), which were shallower but had a higher probability compared with the impaction locations of the other shapes. Unlike other shapes, the large half of the spindle-shaped cases impacted more frequently in the upper esophagus compared with those of the small half (16.14 cm and 15.89%; 16.22 cm and 13.85%, respectively), but the small half predominated in the middle esophagus (24.57 cm, 3.60%). Of the irregular shapes (Fig. 2f), most cases impacted at 19.36 cm (9.10%). In the upper esophagus, however, the large half of the irregular cases impacted less than those of the small half (19.23 cm and 7.07%; 18.84 cm and 9.73%, respectively), and the impaction probability reversed in the lower esophagus. There were not enough cases for the sphere group to obtain a statistically reliable result. Thus, the impaction probability was the highest in the upper esophagus and the lowest in the lower esophagus, the peak densities of impaction were associated with the physical stenoses. The impaction trend for all shapes of different sizes was similar to that of the total cases, with the exception of the sheet-shaped cases. For sheet-shaped cases, more EFBs were impacted deeper, especially in the large half. It was noted that the impaction location was shallower, and the probability of EFB impaction was the highest in the upper esophagus in the spindle shapes compared with other shapes. Figure 3 shows six typical cases that ingested diverse types and shapes of EFBs that were impacted at different locations.

### Management for each shape

Ultimately, 266 cases (62.30%) were treated by FE, 149 (34.89%) by RE, 11 (2.58%) by surgery, and one (0.23%) by bronchoscopy. In addition to the main management, a covered artery stent was placed in the artery near each EFB’s impaction position to prevent major bleeding. In our study, eight cases were treated by covered artery stents before the main retrieval procedure, with the DFI ranging from 22–28 cm, and the distances between each EFB and the aortic wall were <0 mm in four cases, <1 mm in two cases, and <2 mm in two other cases. Four of the eight cases ingested pin-shaped EFBs, three ingested sheet-shaped EFBs, and one ingested an irregular-shaped EFB.

Spindle-shaped EFBs were most commonly removed by RE; the second-line therapy for EFBs. The other five EFB shapes were primarily treated by FE, the most common first-line therapy for EFBs. It seems that only spindle-shaped EFBs required more advanced treatment. However, 9 of all 11 cases treated by surgery were pin-shaped EFBs, and only one case treated by bronchoscopy was a pin-shaped EFB (Table 2). As such, the pin-shaped EFB also required more advanced management. Hence, we speculate that pin- and spindle-shaped EFBs are likely to be the most difficult to treat.

**Table 2:** Managements and complications for different shapes of EFBs.

Shape	Managements						Complications					
	FE (n = 266)	RE (n = 149)	Surgery (n = 11)	Bronchoscopy (n = 1)	χ^2^	*P*	Perforation (n = 86)	χ^2^	*P*	Severe complication (n = 39)	χ^2^	*P*
**Pin (n = 161)**	109	42	9	1	17.724	0.001**	40 (24.84%)	3.556	0.059	17 (10.56%)	0.633	0.426
Fish bone	83	28	9	1	4.984	0.173	33	1.538	0.215	13	0.018	0.894
Poultry bone	22	11	0	0	3.42	0.331	5	2.089	0.148	3	0.095	0.758
Pork bone	2	2	0	0	1.344	0.719	1	<0.001	0.994	1	0.906	0.341
Other animal bone	2	1	0	0	0.252	0.969	1	0.118	0.731	0	0.361	0.548
**Sheet (n = 97)**	63	34	0	0	3.670	0.299	13	3.543	0.060	2 (2.06%)	7.563	0.006**
Fish bone	26	11	0	0	0.744	0.388	5	0.002	0.980	1	0.122	0.727
Poultry bone	28	13	0	0	0.349	0.555	3	2.266	0.132	0	1.495	0.221
Pork bone	6	9	0	0	4.851	0.028	4	2.690	0.101	1	1.863	0.172
Other animal bone	1	1	0	0	0.200	0.654	1	2.357	0.125	0	0.043	0.836
plastic	1	0	0	0	0.545	0.460	0	0.156	0.693	0	0.021	0.884
Other	1	0	0	0	0.545	0.460	0	0.156	0.693	0	0.021	0.884
**Trident (n = 51)**	37	14	0	0	3.525	0.318	7 (13.73%)	1.482	0.223	3 (5.88%)	0.738	0.390
**Spindle (n = 66)**	27	38	1	0	17.764	<0.001***	21 (31.82%)	6.619	0.010*	11 (16.67%)	5.338	0.021*
**Irregular (n = 46)**	27	18	1	0	0.525	0.913	5 (10.87%)	2.755	0.097	5 (10.87%)	0.187	0.665
**Sphere (n = 6)**	3	3	0	0	0.717	0.869	0	1.535	0.215	1 (16.67%)	0.416	0.519

FE: flexible endoscopy; RE: rigid endoscopy. *P < 0.05; **P < 0.01; ***P < 0.001.

The different types of pin-shaped EFBs were also analyzed to determine whether the type had an impact on management. No significant differences existed in each type; thus, the shape, rather than the type, was the factor that determined the treatment. For spindle-shaped EFBs, only cases involving jujube pits were enrolled, so the impact of the type on the management could not be distinguished.

### Complications associated with each shape

**Figure 4:**
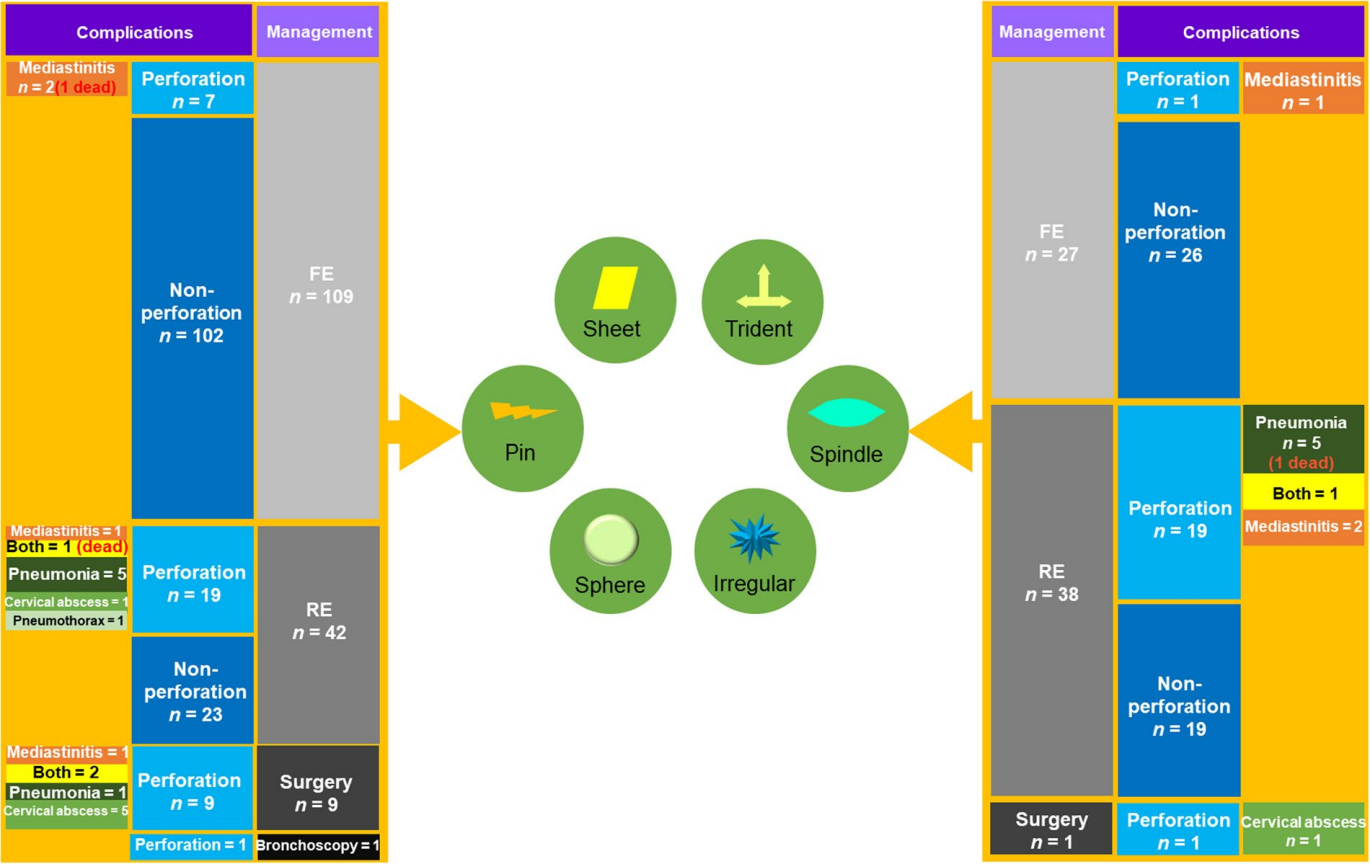
Management and complications for pin and spindle groups. FE: flexible endoscopy; RE: rigid endoscopy; both indicate the patient suffered from both pneumonia and mediastinitis.

Esophageal perforation was detected in 86 cases (20.14%), all of which were traumatic perforation caused by EFBs, as suggested on the CT scan taken prior to removal. Thirty-eight cases were complicated by other severe complications; more seriously, five cases resulted in death. Of the 48 cases of perforation without severe complications, 9 were treated by FE (two cases were closed by clips), and 39 were treated by RE. After removing the EFBs, limiting oral intake by a nose–jejunum nutrition tube or stomach tube, and administering parenteral antibiotics, the perforations were successfully managed, as evidenced by a normal reexamination through CT or a contrast esophagram. Of the 38 cases with severe complications, 4 were treated by FE, 22 by RE, 11 by surgery (6 by the lateral cervical extraction of EFBs, debridement, and drainage; 5 by thoracotomy, primary repair, and drainage), and one by bronchoscopy because the EFB was inserted into the trachea; antibiotic therapy and limiting oral intake were also implemented after the procedure. However, 16 cases had pneumonia (two died of severe pneumonia), 7 had mediastinitis (1 died), 6 had both pneumonia and mediastinitis (2 died), 6 had a cervical abscess, and 3 had a mediastinal abscess, asthma, and pneumothorax. There was only one case that had no obvious perforation and was treated by RE but died of disseminated intravascular coagulation. The management and corresponding complications of the possibly troublesome pin- and spindle-shaped EFBs are shown in Fig. 4. Severe complications often occurred in the cases of perforation treated by RE or surgery.

Table 2 shows the influence of different EFB shapes on the prognoses of cases that ingested said EFBs. The spindle group had the highest percentage (31.82%) of cases suffering esophageal perforation compared with any other group (χ^2^ = 6.619, *P* = 0.010). Similarly, the spindle group had the highest percentage (16.67%) of cases suffering severe complications compared with the non-spindle groups (χ^2^ = 5.338, *P* = 0.021). The pin group had a high percentage (24.84%) of cases suffering esophageal perforation as well, despite no significant difference existing between the pin and non-pin groups. The sheet group had the lowest percentage (2.06%) of cases suffering severe complications when compared with the non-sheet groups (χ^2^ = 7.563, *P* = 0.006). The types in the sheet group did not differ significantly when it came to severe complications, suggesting that shape, rather than type, was still the major concern. Although the sphere group suffered severe complications at a rate identical to that of the spindle group, the data is insufficient to be clinically valuable because there were only six cases in this group.

The cases with perforation all resulted from the ingestion of sharp EFBs (86/409) and no blunt EFBs (0/18) led to perforation (χ^2^ = 4.739, *P* = 0.029). Thirty-eight cases with perforation accompanied by severe complications resulted from sharp EFBs; however, the only case that died of DIC ingested a blunt EFB (χ^2^ = 0.290, *P* = 0.590).

### Clinical features and risk for EFBs based on the number of pressure points

Although most EFBs are sharp regardless of their shape (except for sphere-shaped EFBs), the results above show that the pin- and spindle-shaped EFBs were more troublesome than other shapes. To stratify the clinical risks associated with each shape, the number of pressure points was proposed to determine the characteristics of EFBs based on shape. All sharp EFBs (409 cases) were divided into two groups according to each EFB’s number of sharp ends. The group with two pressure points included the pin- and spindle-shaped EFBs, while the group with ≥3 pressure points was comprised of sheet-, trident-, and irregular-shaped EFBs. As shown in Table 3, the EFBs with two pressure points were significantly impacted in more female patients and older patients in addition to correlating with longer DOIs and shorter DFIs. Although the Lmax was slightly smaller in this group, the Lmin was significantly smaller, so the Lmax/Lmin ratio was significantly higher, which was another parameter reflecting the higher pressure on the esophageal wall.

**Table 3:** Clinical features and risks associated with EFBs based on the number of pressure points.

Parameter	2 pressure points (225 cases)	≥3 pressure points (184 cases)	t or χ^2^	*P*
Gender (male/female)	(79/146)	(100/84)	15.220	<0.001***
Age (y)	55.88 ± 15.66	47.72 ± 18.96	−4.681	<0.001***
DOI (h)	31.33 ± 49.76	22.38 ± 37.12	−2.083	0.038*
DFI (cm)	19.78 ± 4.91	20.98 ± 4.60	2.538	0.012*
LOS (day)	2.69 ± 2.98	2.40 ± 3.84	−0.850	0.396
Lmax (cm)	20.99 ± 8.18	23.07 ± 9.82	2.329	0.020*
Lmin (cm)	4.45 ± 2.38	14.32 ± 6.45	19.693	<0.001***
Lmax/Lmin	5.73 ± 3.18	1.96 ± 1.49	−15.818	<0.001***
**Managements**			7.141	0.068
FE (%)	135 (60.00%)	121 (65.76%)		
RE (%)	79 (35.11%)	62 (33.70%)		
Surgery (%)	10 (4.44%)	1 (0.54%)		
Bronchoscopy (%)	1 (0.44%)	0 (0.00%)		
**Complications**				
Perforation (%)	61 (27.11%)	25 (13.59%)	11.149	0.001**
Severe complication (%)	28 (12.44%)	10 (5.43%)	5.901	0.015*

A group of 2 pressure points contains sharp EFBs of pin-like and spindle shapes; a group of ≥3 pressure points contains sharp EFBs of sheet, trident, and irregular shapes. Blunt EFBs were not included in this table. DOI: duration of impaction; DFI: distance from incisor; LOS: length of stay; Lmax: length of major axis; Lmin: length of minor axis. *P < 0.05; **P < 0.01; ***P < 0.001.

Managements for the patients in each group were not statistically significant; however, the percentage of those treated by FE was lower in the group with two pressure points, while advanced managements, such as RE, bronchoscopy, and, especially, surgery, were higher in this group. Rates of perforation and severe complications were significantly higher in the group with two pressure points. These data not only demonstrate the clinical features of this group but also indicate that EFBs with two pressure points are associated with higher clinical risk, which may explain why pin- and spindle-shaped EFBs are more dangerous than sheet-, trident-, and irregular-shaped EFBs.

### Risk factors for esophageal perforation and severe complications based on binary regression analysis

The binary regression analysis showed that the age, DOI, Lmax, Lmax/Lmin ratio, and spindle shape are independent risk factors of esophageal perforation. With the exception of the Lmax/Lmin ratio, they are also independent risk factors of severe complications (Table 4). Thus far, we have found that spindle- rather than pin-shaped EFBs indicate a higher risk of perforation and severe complications despite that those EFBs consisted of two pressure points.

**Table 4:** Outcomes of binary regression analysis for poor prognosis (perforation and severe complications).

	Perforation				Severe complications			
	B	S.E	Exp(B)	P value	B	S.E	Exp(B)	P value
Gender	0.473	0.319	0.859–2.998	0.138	0.389	0.411	0.659–3.305	0.44
Age	0.024	0.010	1.005–1.044	0.014*	0.053	0.014	1.026–1.083	<0.001***
DOI	0.025	0.004	1.016–1.034	<0.001***	0.009	0.003	1.003–1.015	0.003**
DFI	0.041	0.030	0.982–1.104	0.174	0.063	0.039	0.987–1.148	0.104
Lmax	0.017	0.021	0.976–1.060	0.423	0.050	0.023	1.004–1.100	0.033*
Lmin	0.071	0.045	0.983–1.171	0.114	0.059	0.057	0.949–1.186	0.301
Lmax/Lmin	0.249	0.080	1.098–1.500	0.002**	0.057	0.099	0.872–1.287	0.563
Sharpness	—	—	—	—	0.639	1.216	0.175–20.527	0.599
Shapes of two pressure points								
Pin	0.509	0.608	0.505–5.472	0.403	1.362	0.908	0.658–23.160	0.134
Spindle	1.273	0.563	1.156–6.746	0.024*	1.666	0.775	1.159–24.146	0.032*

DOI: duration of impaction; DFI: distance from incisor; LOS: length of stay; Lmax: length of major axis; Lmin: length of minor axis *P < 0.05; **P < 0.01; ***P < 0.001. The sharpness was not included in the regression analysis for perforation, because all 18 blunt cases were in the non-perforation group.

## Discussion

In adults, most ingested foreign bodies pass spontaneously^1,17^; however, 10–20% of EFB ingestion cases require medical intervention^8^. Previous studies focused on the impaction site, type of EFB, chief complaints, DOI, management, and complications^18^. Few studies divided EFBs into different shapes, such as sharp or blunt EFBs^19^; when they did so, such classification was general and inconsistent. Furthermore, Kim^20^ found that a foreign body with a sharp polygonal or pin-like pointed structure can perforate or tear the esophageal wall. Nevertheless, the characteristics of EFBs correlated with clinical risk based on shape have not previously been demonstrated. In the present study, we not only classified EFB types but also provided novel categories based on EFB shape and introduced the concept of pressure points to stratify the risks associated with each shape.

For the last several decades, numerous types of EFBs have been presented in clinics, such as bones, coins, and pills. However, the EFB type varies according to the dietary habits and sociocultural features of a community. Thus, the proportion of food-related impaction at a given institution may correlate with the specific diet of the nearby population^21^. Interestingly, our study found that jujube pits are frequently ingested in our community, but the shape of the jujube pit rarely appears in clinical studies; thus, we defined the jujube pit as spindle-shaped, i.e., as having an oval body with two sharp points. Other cases were divided into five shapes. Clinical features, such as gender, age, DOI, and DFI, were analyzed for each shape to determine their differences and draw physicians’ attention to those associated with high risk. Considering that previous studies have analyzed size exclusively according to the largest diameter^22^, which is not sufficient to define the configuration of an object, the Lmin was included in our study so that the Lmax/Lmin ratio could define the thinness of the ingested object.

The impaction locations of EFBs were closely linked with the three physical stenoses. Probability density of impaction of all shapes, except the sheet shape, manifested a step-down trend, which confirmed that most EFBs impact at the upper and middle esophagus, regardless of size. However, the large sheet-shaped EFBs tended to impact lower in the esophagus than the small EFBs, but according to the regression analysis, DFI was not an independent factor of poor prognosis.

Regarding the procedures for removing EFBs, RE was generally considered more suitable for removing EFBs located near the esophageal entrance, and FE was more suitable for other areas of the gastrointestinal tract as well as the stomach and duodenum^23,24^. However, additional factors were involved in the selection of RE. For example, elderly patients have more difficulty enduring the process of FE, so RE, which requires general anesthesia, is a better treatment for these patients. Our research statistically verified that only spindle cases are more commonly treated by RE, while others are likely to be treated by FE. However, pin-shaped EFBs, especially those with sharp-pointed ends, had a high risk of penetrating adjacent tissues, such as the aorta^25^, and causing major bleeding; in our study, 9 in 11 cases treated by surgery were pin-shaped; thus, this shape still requires significant clinical attention. There was no significant difference regarding the management of severe complications in each type in the pin or sheet group, which reminded us that the shape of the EFB may be a more important factor of perforation than type. Regarding spindle-shaped EFBs, numerous other types of EFBs with this shape that are smaller or larger than jujube pits exist in nature; however, no such cases were observed in this study, which made it difficult to distinguish the effects of the spindle shape from those of jujube pits specifically.

Prior to the removal procedures, eight cases were treated by covered artery stents because of suggested aortoesophageal injury, half of which had ingested sharp pin-shaped EFBs. These EFBs were all located near the aorta (DFIs ranged from 22–28 cm), which was an important consideration for stent therapy. However, the crucial problem was whether the EFBs had penetrated the aorta. Wei^26^ proposed a classification: a distance of ≤2 cm between the EFB and the aortic wall indicated potential aortoesophageal fistula. In this study, this distance was less than 2 mm in all eight cases. As such, the distance between the EFB and the aortic wall became the decisive factor of aortoesophageal injury.

Generally, esophageal perforation due to foreign body ingestion is rare: it accounts for 1%–4% of the total reported cases^27^. Sharp EFBs have always indicated a high risk of perforation, and the results in this study are evidence of this. However, all blunt cases fell into the non-perforation group; whether sharpness was an independent factor of perforation could not be analyzed by regression analysis in this study. Even for sharp EFBs, the clinical risks differed depending on EFB shape. In this study, spindle-shaped EFBs indicated a significantly higher rate of perforation than any other shape. Although all the perforation cases in this study were diagnosed before any procedures were performed, perforation can still occur following a procedure, which is a chicken-egg problem. The complication rate is higher with RE (10%) than with FE (5%)^28^ because RE has a large working channel with stronger grasping possibilities^29^ and this is more frequently used by physicians to treat severe cases. Thus, the higher rate of perforation in the spindle group might be due to the EFB itself or the frequency with which RE is used to remove EFBs. In addition to advanced managements and a high perforation rate, the spindle group also had the highest rate of severe complications. However, sheet-shaped EFBs had significantly lower rates of severe complications. Therefore, there must be an underlying characteristic linked to the different clinical risk factors of each shape.

The concept of pressure points was proposed in this study to explain and stratify the risks associated with each shape. As we know, P (Pressure) = F (Force)/S (Area). When an EFB is ingested, the esophageal wall exerts a force that pushes against the EFB. The contact area is negatively correlated with the pressure on the area, which explains why sharp EFBs with a small contact area easily result in perforation. Considering the accurate contact area was difficult to measure, the number of pressure points, which was positively correlated with the size of the contact area, became an important parameter. As fewer points represent a smaller area, the pressure is greater and is associated with more risk of perforation and even severe complications. For sharp EFBs, the group with pressure points was treated by more advanced managements and had a significantly higher rate of perforation and severe complications compared with the group with ≥3 pressure points. These results distinguished the risks associated with the pin- and the spindle-shaped EFBs from those associated with sheet-, trident-, and irregular-shaped EFBs and elucidated why the former was more troublesome, while sheet-shaped EFBs led to fewer complications. Blunt EFBs were excluded because the force was distributed across surfaces with significantly larger areas and numbers of points.

Table 2 showed that the spindle shape was associated with a higher risk of poor prognosis compared with the pin shape. Even in the binary regression analysis where the age, DOI, and Lmax were independent factors of severe complications, the spindle shape, in which the Lmax was shorter than in any other case, was still an independent factor for a poor prognosis despite the size. Moreover, the Lmax/Lmin ratio played an important role in inducing perforation; the larger the ratio, the more probable the perforation. As such, sharp EFBs with two pressure points, which had larger ratios, might tend to result in perforation.

The analysis in this study gave us new insight into the predictors of the diagnosis and management of EFB impaction. Previous studies and guidelines have consistently focused on the type, size, duration, and location of EFBs, but we proposed a new parameter–the shape of EFBs–although the shapes in this study did not include all of those that exist in nature. We also provided a novel characteristic–the number of pressure points–to stratify the risk associated with each shape to help physicians quickly diagnose perforation and predict the prognosis.

This study does have some limitations. As a retrospective study, our study only enrolled 427 cases from a single research center; it also only involved patients from one region, so the eating habits of these patients only reflect southern Chinese customs. Moreover, the six shapes we presented are not exhaustive of all shapes, sub-classifications must still be defined. The spindle group consisted only of jujube pits, which made it difficult to distinguish the effect of the shape from that of the type. Only 18 cases ingested blunt EFBs, and all of them were in the non-perforation group, which makes it difficult to determine whether blunt EFBs are an independent risk factor of perforation through regression analysis.

## Conclusions

Our findings suggest that different shapes of EFBs have different characteristics, which can have a remarkable impact on the clinical features, management, perforation rate, and severe complications of EFBs. EFBs with two pressure points, especially spindle-shaped EFBs, are more dangerous compared with those with more pressure points.

## Data Availability

The datasets used and analyzed during the current study are available from the corresponding author on reasonable request.
